# Dr. Hamilton’s Fracture Tables

**Published:** 1853-08

**Authors:** 


*Dr. Hamilton's Fracture Tables.*—An enlarged edition of these very valuable tables, is for sale at Phinney's book-store. A supplement to them has been added, under the supervision of Dr. John Boardman, A. B. The price is a mere trifle; and we trust that our readers will avail themselves of the opportunity of procuring them in a convenient form. In a note addressed to us, Dr. Hamilton speaks as follows, of his motives in collecting these facts:

"My sole purpose in publishing them is to correct, if possible, the tide of prosecutions against surgeons. These cases are collected from the practice of surgeons, in and out of hospitals; both here, and in Europe. Many of them are my own cases.

"In no instance has a case been admitted which was not treated by a regular surgeon; and I have myself measured every limb carefully. The result is, as you will see; that surgeons are far from making limbs perfect after fractures; and the inference must be that our art is still defective.

"I may hope, therefore, that the publication of these 'tables' may lead to some improvements in this branch of surgery." S. B. H.

